# Macrophages in Organ Transplantation

**DOI:** 10.3389/fimmu.2020.582939

**Published:** 2020-11-30

**Authors:** Farideh Ordikhani, Venu Pothula, Rodrigo Sanchez-Tarjuelo, Stefan Jordan, Jordi Ochando

**Affiliations:** ^1^ Department of Oncological Sciences, Icahn School of Medicine at Mount Sinai, New York, NY, United States; ^2^ Immunología de Trasplantes, Centro Nacional de Microbiología, Instituto de Salud Carlos III, Madrid, Spain

**Keywords:** macrophages, immune tolerance, trained immunity, organ transplantation, nanotherapy

## Abstract

Current immunosuppressive therapy has led to excellent short-term survival rates in organ transplantation. However, long-term graft survival rates are suboptimal, and a vast number of allografts are gradually lost in the clinic. An increasing number of animal and clinical studies have demonstrated that monocytes and macrophages play a pivotal role in graft rejection, as these mononuclear phagocytic cells recognize alloantigens and trigger an inflammatory cascade that activate the adaptive immune response. Moreover, recent studies suggest that monocytes acquire a feature of memory recall response that is associated with a potent immune response. This form of memory is called “trained immunity,” and it is retained by mechanisms of epigenetic and metabolic changes in innate immune cells after exposure to particular ligands, which have a direct impact in allograft rejection. In this review article, we highlight the role of monocytes and macrophages in organ transplantation and summarize therapeutic approaches to promote tolerance through manipulation of monocytes and macrophages. These strategies may open new therapeutic opportunities to increase long-term transplant survival rates in the clinic.

## Introduction

Organ transplantation is a life-saving strategy for thousands of patients with end-stage organ failure. Patients who find a compatible donor and receive a transplant are treated daily with multi-drug combinations designed to prevent rejection of the transplanted organ. Thanks to great progress in surgical techniques and immunosuppressive drugs, the percentage of short-term allograft rejection events has declined and 1-year allograft survival rates are above 90% (1). However long-term graft survival rates remain suboptimal (2, 3), arguing in favor of additional mechanisms of immune regulation associated with chronic allograft rejection that escape current immunosuppressive therapy.

**Figure 1:**
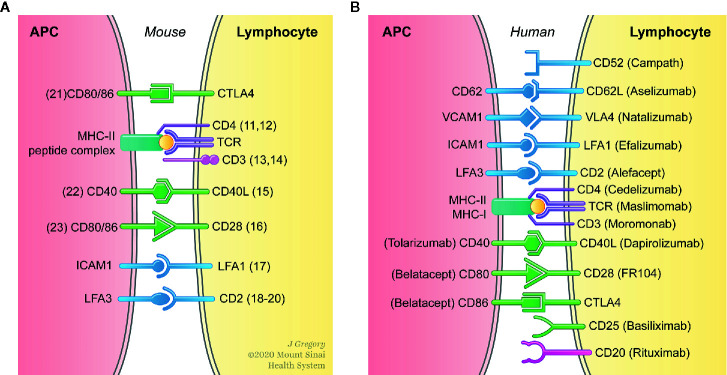
Interfering with binding of the TCR to antigenic peptide complexed with MHC (signal 1) and engagement of co-stimulatory molecules (signal 2) prevents T cell activation. **(A)** Prolonged allograft survival and induction of tolerance has been achieved in experimental animal models buy targeting signals 1 and 2 in both T cells and antigen presenting cells (APC). **(B)** The clinical translation of therapeutic approaches that specifically target APC *in vivo* remain largely elusive.

To promote long-term organ transplant survival in the absence of chronic immunosuppressive therapy, transplant immunologists have historically focused on targeting the adaptive immune response. This is in response to early work on allograft rejection, which demonstrated that T cells are both necessary and sufficient for allograft rejection (4, 5). More recent work has focused on developing novel tolerogenic protocols that target the adaptive immune response using methods that include depletion of effector T cells (6), induction of CD4^+^CD25^+^Foxp3^+^ regulatory T cells (7) and blockade of co-stimulatory signals (8). The latter was achieved using monoclonal antibodies (mAb) or immunoglobulins (Ig) against cell surface molecules (CD4 (9); CD4 + DST (10); CD3 (11); non-depleting CD3 (12); CD40L (13); CD40L + CD28 (14); LFA-1^+^ + ICAM-1 (15); CD2 (16); CD2 + CD3 (17); LFA3-Ig (18); CD80 and CD86 (19); CD40 (20); and CTLA4-Ig (21) (**Figure 1A**). While promising results have been obtained using these therapeutic approaches in experimental animal models, translation of these tolerance promoting methodologies that target innate immune cells in the clinic remain largely elusive (**Figure 1B**). Considering that consistent induction donor specific unresponsiveness remains a difficult task in the clinic, there is a major unmet need for the development of additional immune regulatory programs to improve long-term allograft survival in the clinical practice. Since innate immune cells participate in allograft recognition, developing therapeutic approaches that target myeloid cells in the clinic could open novel avenues to improve long-term transplantation outcomes.

It is widely accepted that allograft rejection is the result of a complex series of interactions between both the innate and the adaptive immune systems (22, 23). Recent advances in our understanding of the mechanisms that determine the outcome of the immune response to transplanted organs have highlighted the importance of the innate immune response (24). This ancient part of the immune system precedes cellular and humoral immunity and consequentially regulates the function of the adaptive immune response. The innate immune response initiates inflammatory signals as a defense mechanism against pathogens and tissue injury. Non-self-inflammatory stimuli induced by exogenous infectious agents are considered pathogen-associated molecular patterns (PAMPs), while tissue injury is recognized by self-derived damage-associated molecular patterns (DAMPs). Both PAMPs and DAMPs are recognized through pattern recognition receptors (PRRs), which include Toll-like receptors (TLR), NOD-like receptors (NLR) and C-type lectin receptors. PRRs are expressed on the cell surface and in the cytoplasm of innate immune cells, including macrophages, and mediate intracellular signaling cascades leading to transcriptional expression of inflammatory mediators (25).

Macrophages belong to the mononuclear phagocyte system and have a dual role in allograft transplantation, either triggering inflammatory response or inducing a tolerogenic environment (26). Local activation of macrophages through PRRs can lead to upregulation of major histocompatibility complex (MHC) and co-stimulatory molecules (signals 1 and 2), as well as the production of pro-inflammatory cytokines (signal 3) which result in T cell proliferation and differentiation (27, 28). More recently, it was demonstrated that macrophages adopt a long-term pro-inflammatory phenotype following an initial PRR stimulation of the C-type lectin receptor dectin-1, which results in a non-specific memory of the innate immune cells mediated by epigenetic reprogramming (29). This novel macrophage functional state has been termed trained immunity and is associated with pro-inflammatory cytokine production (TNFα and IL-6) after a second PRR stimulatory signal with TLR4 agonists (30). Understanding the immune biology of trained immunity has important implications for the design of novel therapeutic approaches. Preventing the accumulation of trained macrophages while promoting the development of regulatory macrophages represents an attractive, innovative approach to promote organ transplant acceptance. Herein, we highlight recent studies on the role of macrophages in organ transplantation and summarize the therapeutic potential of targeting macrophages for the induction of tolerance.

## Macrophage Heterogeneity and Plasticity

Monocytes and macrophages are key elements of innate immunity and have crucial roles in host defense, inflammation and tissue homeostasis (31, 32). Monocytes originate from myeloid progenitor cells in the bone marrow and circulate in the blood for several days before entering the tissue and differentiating into macrophages (33, 34). Monocyte-derived macrophages also have key roles in clearing pathogens and cell debris, antigen presentation and initiating adaptive immune responses (35). To do so, macrophages acquire specialized functions according to the stimuli present in the environment. In relation to their activation, Mills et al. proposed two phenotypes: classical (M1) *versus* alternative (M2), in analogy to T helper cells Th1 and Th2 (36, 37). M1/M2 macrophages are functionally distinct with M1 macrophages shifted to nitric oxide (NO) and citrulline secretion, while M2 macrophages shifted toward production ornithine and polyamine secretion (36, 37). Consequentially, M1-derived NO inhibits T cell proliferation and exhibits a potent microbicidal activity, while M2-derived ornithine promotes cell proliferation and repair through polyamine and collagen synthesis (38–40). Over the past few years, this nomenclature has been a matter of debate due to the difficulty of including within M1 and M2 classification the multiple phenotypes adopted by macrophages. While *in vitro* activation of macrophages allowed us to better understand the developmental requirements of different macrophage subsets, *in vivo* studies are more complicated because the stimuli they encounter are multiple, complex and occur simultaneously (31, 41, 42).

**Figure 2:**
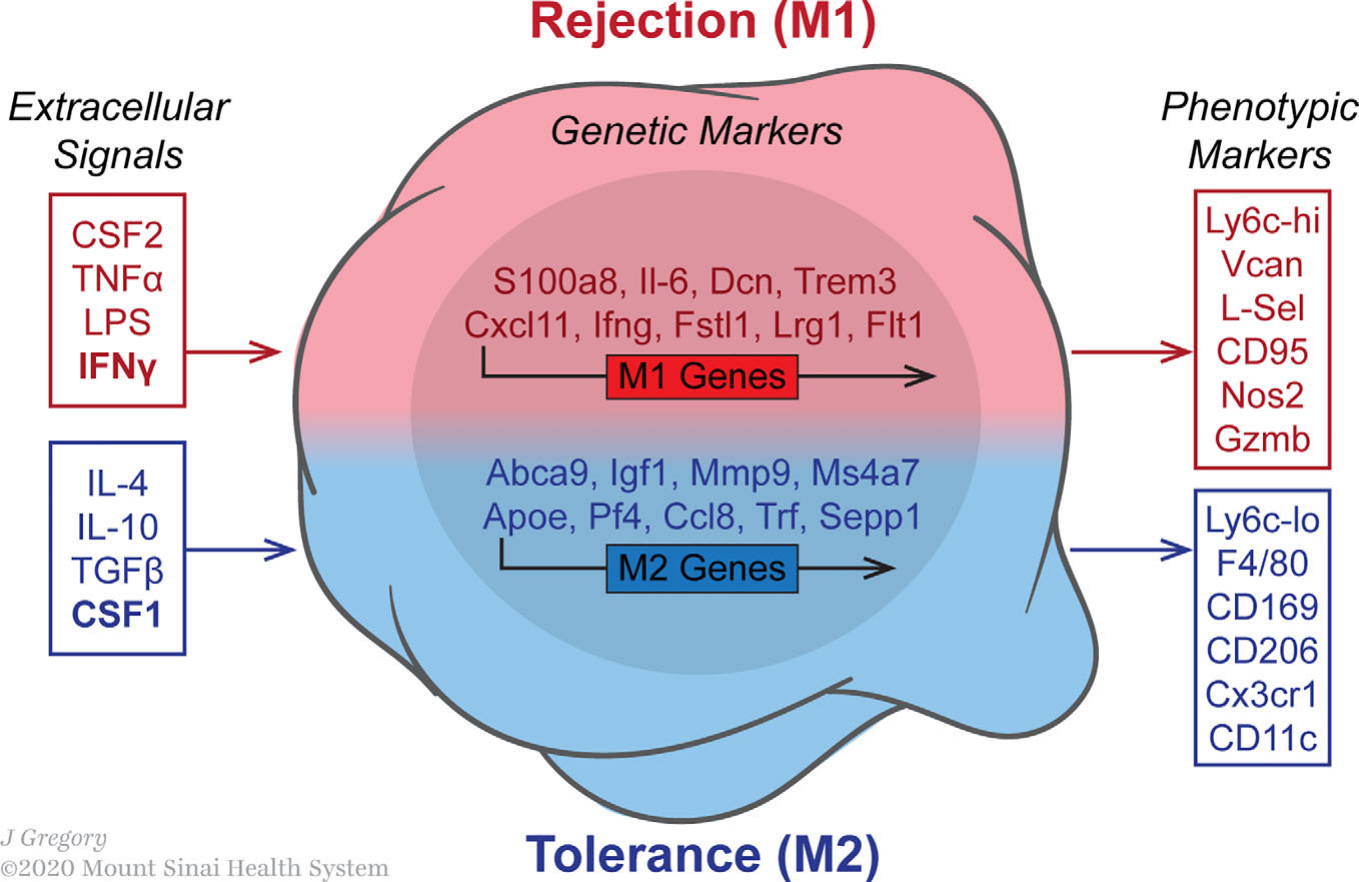
Macrophages polarization in the transplanted allograft is influenced by various stimuli. The image indicates the expression of macrophage genes encoding cytokine receptors, cell activation markers and cell adhesion molecules. Data was acquired from published microarray obtained from graft-infiltrating macrophages on day 5 post-transplantation in either untreated rejecting or anti-CD40L mAb treated mice. The GEO accession number for the microarray data reported in this figure is GSE68648.

Various stimuli control the expression of macrophage genes encoding cytokine receptors, cell activation markers and cell adhesion molecules **(** **Figure 2** **)**. Classic or M1 macrophage activation increases in response to PAMPs, DAMPs and pro-inflammatory cytokines such as interferon-γ (IFN-γ) and tumor-necrosis factor (TNF) (43, 44). The environment favors the production of inflammatory chemokines by M1 macrophages, which induce lymphocyte recruitment. Among the chemokines produced by these M1 macrophages are CXCL9 and CXCL10, strongly associated with Th1 immune response (45, 46) and CXCL16, which maintain M1 polarization (47). Upon activation, M1 macrophages produce high levels of pro-inflammatory cytokines such as TNF, IL-1β, IL-6, IL-12 and IL-23, which may result in functional CD4^+^ T lymphocyte polarization toward Th1 (48–51) or Th17 (52–54). In addition, M1 macrophages produce high levels of inducible nitric oxide synthase 2 (iNOS2) and reactive oxygen intermediates (ROI) that participate in removing bacteria, viruses and parasites. Phenotypically, costimulatory molecules such as CD40, CD80 and CD86, important in antigen presentation, are upregulated in M1 macrophages in conjunction with major histocompatibility complex class II (MHC-II) (55–57).

In contrast, M2-polarized macrophages, also known as alternatively activated macrophages, are important in tissue repair. The M2 phenotype contains different macrophage populations with separated functions, which can be polarized by several stimulatory factors. Based on the stimuli and transcriptional changes, Mantovani and Rőszer divided the M2 phenotype into M2a, M2b, M2c and M2d subtypes (58, 59). The mutual characteristics of these subtypes are high secretion of IL-10 and low IL-12 levels, in conjunction with the generation of arginase-1 (Arg-1). M2a macrophages are induced by IL-4 and IL-13, express high levels of mannose receptor (CD206) and secrete pro-fibrotic factors, such as TGF-β, to contribute towards tissue repair (60–62). M2b macrophages have phenotypical and functional similarities with regulatory macrophages. They are activated by TLR or IL-1R agonists and produce both pro and anti-inflammatory cytokines, such as TNF-α, IL-1β, IL6 and IL-10 (41, 63). M2c macrophages, also known as inactivated macrophages, are induced by IL-10 and display anti-inflammatory functions. M2c secrete IL-10 and TGF-β (59, 64) and are efficient at phagocytosis and elimination of apoptotic cells (65). M2d macrophages have phenotypical and functional similarities with tumor-associated macrophages (TAMs). They are induced by A2 adenosine receptor (A2R) and IL-6 (66–68) and secrete IL-10, TGF-β and vascular endothelial growth factor (VEGF) to favor angiogenesis and cancer metastasis (68–70).

The need to update the M1/M2 classification has been evidenced in numerous studies addressing signaling pathways and genetic signatures associated with M1/M2 polarization (71–75). M1 and M2 share many genes implicated in cellular functions, such as phagocytosis, metabolism and cytokine production. IL-8, Tissue Factor and Leukocyte extravasation signaling pathways are shared among M1 and alternatively activated M2 (76, 77). On the other hand, recent works show specific signatures for M1 and M2 (78). For example, Jablonski et al. identified a new set of common and distinct M1 and M2 macrophage genes. They showed that CD38, Gpr18 and Fpr2 were M1-specific while c-Myc and Egr2 were M2-specific genes, proposing a new way to define both states of polarization based on their phenotypes: CD38^+^ Egr2^−^ (M1 macrophages) and CD38^−^ Egr2^+^ (M2 macrophages) (71). In addition, Buscher et al. demonstrated a strong gene-environment interaction in activated macrophages using a hybrid mouse diversity panel (HMDP). They showed different genetic signatures associated with lipopolysaccharide (LPS) responsiveness among a wide spectrum of macrophage phenotypes from several different inbred strains (72). Recently, Orecchioni et al. compared both transcriptomes obtained from Jablonski (*in vitro*) and Buscher (*in vivo*) to define differential signatures present in M1/M2 macrophages (79) and concluded that Fcγ receptor-mediated phagocytosis, MAPK signaling, MAPK, JAK1 and JAK3 signaling are upregulated in M1 upon LPS activation. These pathways control several inflammatory genes that allow the macrophages to exhibit their pro-inflammatory properties (80, 81). In contrast, the main pathways specifically expressed in M2 are adipogenesis, fatty acid synthesis and integrin signaling pathways, which are important for tissue infiltration, removal of necrotic tissue and initiation of tissue regeneration (82).

While bone marrow monocytes are mobilized early after transplantation and recipient monocyte-derived macrophages represent the majority of macrophages in the transplanted organ (83), it is important to acknowledge the immune regulatory role of tissue resident donor macrophages. Tissue-resident macrophages (TRMs) arise from fetal liver or yolk-sac progenitors and are phenotypically distinct from monocyte-derived macrophages in steady state conditions (84). While TRMs are primarily characterized by the expression of CD11b, F4/80, CD64, CD68 and MerTK and low levels of MHC-II on the cell surface in mice, monocyte-derived macrophages are characterized by CD11b, CD209, CD64 and MerTK expression on the cell surface (85). TRMs are functionally considered to be immunosuppressive because of their fundamental roles in maintaining homeostasis, inhibiting T cell activation and promoting the resolution of inflammation (75, 86). TRMs are divided into subpopulations according to their anatomical sites and functionality. For instance, Kupffer cells in liver (87, 88) or alveolar macrophages in lung (89) exhibit critical roles in generating CD4 regulatory T cells (Treg) and promoting tolerance. In the context of organ transplantation, Terry Strom and colleagues identified a subset of donor TRM that express high levels of the phosphatidylserine receptor TIM4 and CD169. The study demonstrated that this population of macrophages migrates to the draining lymph nodes following oxidative stress during ischemia-reperfusion injury (IRI) associated with transplantation and induces antigen-stimulated Treg. Interestingly, these M2-like TIM-4+CD169+ donor TRM were demonstrated to be immunoregulatory and to promote the engraftment in a murine cardiac allograft model (90). Contrary to this view, it has been suggested that ischemia/reperfusion primes innate immune cells for an excessive response to a subsequent inflammatory, which promotes organ injury. In the lung, alveolar macrophages under shock/resuscitation events increase their TLR4 expression in the cell surface due to oxidative stress (91). As a result, alveolar macrophages are primed and exhibit an exaggerated LPS response following a secondary stimulation. The source of the endotoxin is not clear, but it has been suggested that LPS may leak from the gut under ischemia/reperfusion conditions (92). This has major implications in lung transplantation as oxidative stress induced during IRI, coupled with an increase in the endotoxin levels in the donor organ is associated with increased neutrophil recruitment as well as physiological markers of allograft injury mediated by tissue resident alveolar macrophages through TLR4/MyD88 dependent pathways (93). Consequentially, presence of endotoxin in the lung predisposes the donor organ to the fatal syndrome of primary graft dysfunction (PGD) and compromises the survival of the allograft following lung transplantation. Overall, the data suggests that while TRMs present in the donor organs may favor immunoregulatory mechanisms that promote allograft engraftment (94), their suppressive activity may be reversed toward a pro-inflammatory functional state (95), compromising organ transplant survival.

### Macrophages and Rejection

Macrophage accumulation has long been recognized as a feature of allograft rejection (96). The total number of graft infiltrating macrophages correlates with worse clinical outcomes (97, 98) and with acute allograft dysfunction in kidney transplant recipients (99). Early studies from Hancock and colleagues demonstrated that macrophages represent the majority of cells that infiltrate an allograft during severe rejection episodes (100). Using immunohistochemical approaches, their study reported that macrophages represent 60% of graft‐infiltrating cells in severe rejection, 52% in mild rejection and 38% in moderate rejection (100). Looking at the patterns of graft-infiltrating cells during the first days after transplantation, various human studies have shown that the initial accumulation of monocytic cells occurs in all grafts (rejecting and non-rejecting) (101) and that infiltration of kidney allografts by macrophages within the first week of transplantation is associated with worser clinical outcomes (102). Similarly, Schreiner et al. showed an initial accumulation of macrophages in the first 24–48 h after transplantation for both donor kidney allografts and isografts, with a marked increase in monocytes/macrophages being observed only in allografts 96 h after engraftment. As such, it is not surprising that depletion of macrophages has been used to attenuate graft injury and decrease inflammation in acute rejection models (103, 104). To this end, Jose et al. by depletion of macrophages with liposomal-clodronate in a renal transplant rat model showed the contribution of macrophages to tissue damage during acute rejection (105). In another study, Ma et al. demonstrated that the depletion of monocytes/macrophages with c-fms kinase inhibitor resulted in less renal allograft dysfunction and structural damage compared to the vehicle-treated rats (106). Data from our laboratory demonstrated early after transplantation that M1-like monocytic precursors leave the bone marrow and infiltrate heart allografts in transplanted mice (107). Importantly, while M1-like monocytes rapidly convert to M2-like regulatory macrophages in the allografts of transplant recipients under costimulatory blockade treatment with anti-CD40L mAb, untreated recipients maintain M1-like inflammatory macrophages in the rejecting allografts (108). Interestingly, depletion of recipient CD11b cells using CD11b-DTR mice as recipients, prevented the induction of tolerance. This suggests that initial events that regulate macrophage polarization (M1 to M2) rather than depletion may control the fate of the immune response, since depletion of macrophages may affect the protective role of wound healing and tissue remodeling macrophages that are required to restore homeostasis in the donor organ after the transplant surgical procedure.

Despite the significant progress in determining the roles of macrophages in acute graft rejection, the mechanisms by which macrophages mediate tissue injury are not completely understood. One of the suggested mechanisms by which macrophages mediate graft loss is through the production of nitric oxide contributing to the endothelial cell cytotoxicity and tubular injury (103). Acute rejection in heart transplant recipients was associated with severe fibrosis in 1-year biopsies, which was associated with higher CD68^+^CD163^+^ M2 macrophages compared to barely present CD68^+^CD80^+^ M1 macrophages in graft (109). Similarly, infiltrating macrophages in renal allograft 1-year after transplantation exhibited an M2 phenotype with CD68^+^ CD206^+^ dual staining (110). It has also been suggested that CD16^+^ monocytes might be responsible for the development of acute allograft rejection after liver transplantation, which may be associated with inhibition of Treg cells (111). Furthermore, whole-genome transcriptome analysis of biopsy samples identified an inflammatory macrophage polarization–specific gene signature, which is upregulated during acute rejection (112). In fact, the degree of macrophage infiltration correlates with increased incidence of allograft rejection (34). Consistent with the increased of macrophage/monocytes infiltration, the level of monocyte colony stimulating factor (M‐CSF), a key cytokine in monocyte recruitment, is elevated in the graft during clinical rejection (113). Moreover, activated monocytes are detectable in the circulation before the clinical symptoms of acute rejection occur (114).

Gradual replacement with recipient-derived macrophages over time leads to chronic rejection through mechanisms that involve cell death, fibrosis, smooth muscle proliferation and cytokine-mediated inflammation (115). Although inflammation is supposed to be short lived and self-limited, acute inflammation can sometimes shift toward a long-lived and self-perpetuating chronic inflammatory response (116). Chronic inflammation develops within months to years after organ transplantation and is the major cause of long-term graft loss (115). The main feature of chronic rejection is obliterative vasculopathy, often accompanied by parenchymal fibrosis which results in ischemia, cell death and progressive graft failure (115, 117). Chronic rejection is characterized by infiltrating T cells and macrophages, although other cellular compartments include natural killer cells, dendritic cells, B cells and plasma cells also play a role in chronic rejection (116). However, the high number of infiltrating macrophages in the allograft, as well as their potential to produce cytokine/growth factor suggests the crucial role of macrophages as end‐effector cells in a final common pathway toward cardiac allograft vasculopathy (CAV) independent of T‐cell or B‐cell alloreactivity (118).

Accumulation of alternatively activated M2-type macrophages is the major macrophage population localized in areas of interstitial fibrosis in chronic kidney allograft injury and correlates with the severity of fibrosis and graft rejection (110, 119). M2 polarization is considered to be anti-inflammatory, immunoregulatory and important for tissue repair and regeneration. However, during chronic rejection, the pro-fibrotic function of M2-polarized macrophages promotes interstitial fibrosis and contributes to graft failure (120). Graft-infiltrating macrophages during chronic rejection are a heterogeneous population expressing markers that are associated with M1 inflammation but also with an M2 immunoregulatory phenotype. It is possible though that immunoregulatory M2 cells are derived from M1 cells in the graft, when the pro-inflammatory microenvironment subsides over time. The predominance of a certain macrophage polarization state in the graft might determine the clinical success of the transplantation. In human kidney transplant recipients, a higher M2 ratio is associated with chronic glomerular injury and poorer graft function (121). Despite the apparent predominant role of M2 macrophages in chronic graft rejection, M1 macrophages might critically contribute with the production of eicosanoids, proteases, ROS and NO (122). To prevent chronic rejection, Liu et al. investigated the effect of macrophage depletion for a certain amount of time in a rat allogenic heart transplant model (123). Their results suggested that macrophage depletion after heart transplantation could alleviate chronic rejection through M2 polarization of regenerated macrophages, as well as the alternation of expression levels of IFN-γ, TNF-α, MCP-1 and IL-10 (123). These approaches deplete macrophages and blocking monocyte recruitment by targeting CCR- and CXCR-mediated chemotaxis that reduce vasculopathy (118, 124, 125).

The granulocyte-macrophage colony-stimulating factor (GM-CSF) and the macrophage colony-stimulating factor (M-CSF) are some of the known factors that regulate differentiation, proliferation, and function of tissue macrophages and determine the outcome of the immune response (126). While GM-CSF induces a state in which macrophages are primed for M1, M-CSF induces M2 macrophage polarization (125, 127). In a recent study, our group elucidated the molecular mechanisms behind CSF-1-mediated macrophages polarization. Our results exhibited that graft‐infiltrating neutrophils in tolerized recipient allografts secreted higher levels of M-CSF compared to neutrophils from untreated rejecting mice, suggesting a potential role of M-CSF producing neutrophils in mediating regulatory M2 macrophage accumulation in the transplanted allograft (128).

Manipulation of M1/M2 polarization represents another therapeutic approach to prevent allograft rejection. Xian Li and colleagues demonstrated that M1/M2 macrophage polarization is dependent on tumor-necrosis factor receptor-associated factor 6 (TRAF6) and mammalian target of rapamycin (mTOR), respectively (129). While mice deficient for TRAF6 in macrophages prevents accumulation of M1 macrophages in recipient mice that develop severe transplant vasculopathy, deletion of mTOR prevents accumulation of M2 macrophages in long-term allograft survival without histological indications of chronic rejection, emphasizing the role of M2-polarized macrophages in chronic allograft rejection (129). The Xian Li laboratory further investigated differences between M1 and M2 macrophages and identified the adenosine triphosphate (ATP)-gated ion channel (P2x7r) as a marker of M2 cells (130). Interestingly, blockade of P2x7r using oxidized ATP, prevented M2 polarization *in vitro* and graft-infiltration *in vivo*, leading to long-term heart allograft survival. This study demonstrated that pharmaceutical targeting of M2 graft-infiltrating macrophages during chronic rejection is a promising strategy to prolong graft survival. Consistent with this view, specific deletion of RhoA or inhibition ROCK kinases with a combination of Y27632, Fasudil and Azaindole inhibited vessel occlusion and tissue fibrosis, decreased M2 macrophage infiltration and abrogated chronic rejection of cardiac allografts (131, 132).

Besides their M1/M2 pro-inflammatory and immunoregulatory functions, it is also possible that macrophages contribute to graft rejection by additional mechanisms. Macrophages in biopsy specimens from patients with active chronic renal allograft rejection co-expressed the macrophage marker CD68 as well as the myofibroblast marker a-smooth muscle actin (a-SMA), suggesting that macrophages undergo a macrophage-to-myofibroblast transition leading to interstitial fibrosis and reduced graft function (133). Similarly, cells co-expressing macrophage and a-SMA markers were found in allografts in mice. These cells derived from recipient bone marrow cells, thus were infiltrating the graft and also co-expressed M2 marker CD206. Further mechanistic studies identified a crucial role for Smad3 in macrophage-to-myofibroblast transition (133).

One key feature of circulating monocytes is their ability to migrate to the inflamed tissue and to initiate the immune response against non-self antigens. Fadi Lakkis and colleagues reported that F4/80^−^Ly6C^+^ neutrophils, F4/80^int^Ly6C^+^ monocytes and F4/80^hi^Ly6C^−^ macrophages rapidly infiltrate sites of inflammation and elicit an allospecific immune response. Remarkably, in contrast to the allogeneic non-self recognition by T cells that recognize MHC molecules, macrophages were shown to recognize non-MHC molecules (134). Using B6-OVA (H-2^b^) and B6F1-OVA (H-2^b/d^) donor heart grafts transplanted into B6 Rag^−^/^−^γc^−^/^−^ (H-2^b^) recipients, this group further demonstrated that only monocytes and DC from B6 Rag^−^/^−^γc^−^/^−^ recipient mice receiving B6F1-OVA (but not B6-OVA) grafts, were able to promote acute cellular rejection upon transfer of OVA antigen-specific CD4^+^ OT-II cells. The Lakkis laboratory, went on to demonstrate that monocytes and macrophages detect the polymorphic molecule signal regulatory protein α (SIRPα) on donor cells to initiate the innate alloresponse (135). SIRPα is a regulatory immunoglobulin superfamily receptor that represents a key member of the “do-not-eat-me” signaling pathway that avoids the to avoid immune response by phagocytes. SIRPα is expressed by myeloid (136) and myeloid-derived suppressor cells (MDSC) that accumulate after organ transplantation and mediate allograft tolerance (137). Mechanistically, engagement of SIRPα with its ubiquitous ligand CD47 delivers inhibitory signals and suppresses the phagocytic function and inflammatory signaling of macrophages (138–140). In the context of organ transplantation, the Lakkis laboratory demonstrated that blocking SIRPα or CD47 with monoclonal antibodies induced graft dysfunction and rejection. Blocking of SIRPα-CD47 interaction results in MDSC differentiation into myeloid cells overexpressing MHC class II, CD86 costimulatory molecule and increased secretion of macrophage-recruiting chemokines leading to loss of tolerance (141). However, a donor allograft with a SIRPα molecule that is mismatched with CD47 leads causes monocytic cell activation and initiation of the immune response to the transplanted organ (135). More recently, the Lakkis laboratory also demonstrated that polymorphisms in the SIRPα gene were required to induce monocyte memory is against non-self MHC molecules. In this study, it was demonstrated that deleting the PIR-A in the recipient or blocking the paired immunoglobulin-like receptor-A (PIR-A) binding to donor MHC-I with a PIR-A3/Fc inhibits alloantigen specific memory of myeloid cells and promotes indefinite allograft survival in a murine kidney and heart transplant model (142). Overall, these studies provide compelling evidence demonstrating that monocytes initiate the immune response, determine the critical role of SIRPα polymorphic differences in the activation of graft reactive macrophages and that the immunological memory to innate myeloid cells can be potentially targeted to promote the induction of transplantation tolerance.

### Macrophages and Tolerance

The participation of graft-infiltrating macrophages in the rapid, stereotypical inflammatory reactions that cause secondary tissue damage during ischemia-reperfusion injury (143) and acute episodes (144) has been long-recognized. However, we are also beginning to understand the vital role of suppressor macrophages in preventing rejection and re-establishment of tissue homeostasis after transplantation (145). Given their influence over transplant outcome, manipulating the balance between graft-protective and graft-destructive macrophage activities represents an attractive therapeutic strategy (146). Various approaches to controlling macrophage responses have been proposed, including adoptive cell therapy with regulatory macrophages (Mregs). In previous work, it was shown that treatment with *ex vivo*-generated CD11b^+^ Ly6C^−/low^ Ly6G^−^ CD169^+^ Mregs could prolong fully-allogeneic heart graft survival in non-immunosuppressed mice (147). Mechanistically, Mregs can directly suppress T cell proliferation and survival through an iNOS-dependent pathway and the secretion of anti-inflammatory factors (148). More recently, Riquelme et al. demonstrated that Mregs induce TIGIT^+^FoxP3^+^ Tregs that produce IL-10 and non-specifically mediates bystander suppression of allo-stimulated CD4+ and CD8+ T cells (149). An equivalent population of human CD11b^+^CD115^+^DC-SIGN^+^ Mregs arises from peripheral blood CD14^+^ CD16^−^ monocytes that are cultured with M-CSF for 6 days prior to stimulation with IFN-γ (150). During this period, a gradual down-regulation of CD14 is observed, which may recapitulate the physiological transition of human M1-like CD14^+^ CD16^−^ inflammatory monocytes into M2-like CD14^−/low^ CD16^+^ resident macrophages. Interestingly, presence of human Mregs correlates with an increase in TIGIT^+^FoxP3^+^ Treg in kidney transplant recipients (149), which is consistent with the preclinical experiments described above. In the clinical setting, Mregs are currently being investigated in humans in the *ONEmreg12* trial, a phase-I/II study to minimize maintenance immunosuppression in kidney transplant recipients (151). This and previous clinical studies suggest Mregs could be used as a cell-based tolerance-promoting therapy, and for this purpose a good manufacturing practice-compliant production process for manufacturing an Mreg-containing cell product, known as “Mreg_UKR,” has been established (152).

Suppressive macrophages are also be generated in recipient mice treated with costimulatory blockade. Our laboratory demonstrated that anti-CD40L mAb favors accumulation of CD11b^+^CD115^+^DC-SIGN^+^ expressing macrophages in the allograft, which promotes the expansion of Treg, while inhibited CD8^+^ T cell accumulation (108). Mechanistically, DC-SIGN macrophages produce regulatory IL-10 and their *in vivo* accumulation is controlled by M-CSF, which is consistent with the Mreg development requirements, phenotype, and function as described by James Hutchinson laboratory above. Besides costimulatory blockade, nanoparticles have also been used to deliver immune regulatory agents to monocytes and macrophages *in vivo* (153). For example, delivery of mycophenolic acid (MPA) by means of PLGA nanoparticles (NP) results in a significant allograft survival prolongation compared to conventional MPA treatment in a murine model of skin transplantation. Mechanistically, Daniel Goldstein and colleagues demonstrated that uptake of NP‐MPA by myeloid cells leads to upregulation of programmed death ligand‐1 (PD‐L1), which results in decreasing their potential to prime alloreactive T cells associated with prolonged allograft survival (154). More recently, our laboratory described a promising strategy to induce long-term allograft survival through *in vivo* targeting of macrophages with nanobiologics. Our laboratory used an effective *in vivo* platform to deliver an mTOR inhibitor (mTORi) and NF-kB inhibitor (TRAF6i) *via* high density lipoprotein nanobiologics (HDL) in a murine vascularized heart transplant model. The HDL-based nanobiologics preferentially targeted myeloid cells and promoted M2 regulatory macrophage polarization, which resulted in prevention alloreactive CD8 T cell-mediated immunity and expansion of Treg (155). As a result, we believe that nanobiologics-based delivery of immunotherapeutic agents has great potential in organ transplantation as they improve the pharmacokinetics, minimize the off-target effects, maximize its dosage at the site of action, and can be as used as controlled release systems in a spatiotemporal manner (156). Taken together, it has become evident that the *in vivo* manipulation of macrophages through the use of nanobiologics represents a promising strategy for long-term allograft survival.

### Epigenetic Regulation of Macrophages and Innate Immune Memory

Macrophages are highly plastic cells that adopt M1 and M2 phenotypes through mechanisms ultimately resulting from integrating their preexisting history and surrounding environmental signals to enable a distinct transcriptional program. In addition, their distinct transcriptional program must enable their phenotype to be distinct from other myeloid cells. The transcriptional program that makes them distinct is controlled *via* various epigenetic processes, among which include DNA methylation, histone modification and expression of non-coding RNAs. These epigenetic modifications of the landscape lead to either compaction or opening of the chromatin, followed by the combination of DNA and DNA-binding proteins, which are associated to gene activation or repression. This is the basis of trained immunity, a new concept in the field, which postulates that innate immune cells can retain a memory of certain primary stimuli *via* epigenetic mechanisms, thus potentially priming them to initiate a stronger response upon a secondary stimulus.

The term “epigenetics” was first pioneered by C.H. Waddington, seeking to explain how phenotypes could be explained not solely by genetic inheritance (157). He later then proposed the concept of the “epigenetic landscape,” which posited that as cells differentiate, they become restricted in their possible fates (158). This concept of the epigenetic landscape was further elaborated on by Thomas Jenuwein and David Allis with their proposal of a “nucleosome code,” an extension of the “histone code” (159, 160). In their “nucleosome code” hypothesis, they propose that certain covalent modifications to the tails of histones in a region of DNA ultimately result in regional compaction or opening of chromatin. How closed or opened the chromatin in a particular region is then ultimately governs the ability of DNA-binding proteins and ultimately RNA Polymerase from binding to certain genes and subsequently transcribing. The histone modifications that encourage opening of the chromatin include H3K4me3, H3K9ac, and H3K27ac, weaken the grip tail of histone 3 (H3) to the DNA allowing other DNA-binding proteins to bind, while repressive histone modifications including H3K9me3, H3K27me3, and H3K36me3 enhance the grip of H3 to the DNA promote the opposite effect. How protected the DNA is by chromatin opening or compaction, as a result of these histone modifications regionally, ultimately mediates the accessibility of RNA Polymerase to specific sites, thus governing gene activation or gene repression.

The link between an external stimulus to macrophages and modification of the epigenetic landscape, thus establishing the importance of the epigenome in macrophages, was first established in 1999, where LPS stimulation was shown to induce IL12 p40 production by the remodeling of nucleosomes positioned at its promoter (161). This process was later shown to be TLR-dependent *via* acetylation of residues on histone 3 and histone 4 typically associated with open chromatin. On a genome-wide level, TLR activation has been shown to induce a program where the “brakes” on inflammatory gene expression are withdrawn by removing repressive histone modifications. Specifically it was shown that the H3K27me3 demethylase JMJD3, is induced by LPS stimulation in macrophages, and thus promotes an inflammatory gene program (162). Conversely, histone modifications pertaining to gene activation, modifications that lessen the grip of nucleosomes on the DNA, are added on at specific loci upon LPS stimulation by various epigenetic writers including histone methyltransferase myeloid lymphoid leukemia (163). The fact that macrophages’ epigenetic architecture is easily changeable upon external stimulation should not be surprising, given that large changes in histone methylation and acetylation patterns occur in the transition from monocytes to macrophages alone (29). In summary, these early studies made it clear that significant epigenetic changes were happening in macrophages.

Prior to stimulation to an exogenous substance, the epigenetic landscape of monocytes and macrophages must be properly established to develop their distinguished phenotype. This is done by the LDTFs (lineage-dependent transcription factor) PU.1 and the C/EBP family of transcription factors, which bind to macrophage-specific genes and enhancers and are critical for proper monocyte and macrophage development (164). These transcription factors are thought to prime these sites, including those of inflammatory genes, suggested by the fact that these loci are marked by the presence of PU.1, H3K4me1, and open chromatin. However, to keep the brakes on the expression of inflammatory genes, these same loci of inflammatory genes are decorated with repressive histone marks that promote chromatin compaction including H3K9me3, H3K27me3, and H4K20me3 and are bound by co-repressors (165–168). Only upon exogenous stimulation, these brakes are released by appropriate epigenetic erasers on the enhancers and promoters of inflammatory genes, and concurrently activating histone marks are added on by appropriate epigenetic writers.

Trained immunity is a relatively new compelling concept in immunology, whose foundation is primarily epigenetic based. It posits that innate immune cells can retain a memory after a primary stimulus and after a return to a resting phase enact a heightened response upon a secondary stimulus (169). The concept was first proposed in 2011 as a means to explain the phenomenon in vertebrates of protective effects of vaccinations or infections, including BCG vaccination and *C. albicans* infection, to unrelated stimuli in a manner independent of the adaptive immune system (170). Soon after, the mechanisms underlying these memory phenomena were soon determined to be based on epigenetic and metabolic reprogramming, with the two being intertwined (29, 171–173). Specifically, significant H3K4me3 deposition upon either BCG vaccination or ß-glucan stimulation was found at the gene promoters of inflammatory genes including TNF-α, IL-6 and glycolysis genes including hexokinase and phosphofructokinase, thus establishing a memory in macrophages. This process was shown to be was mTOR-dependent (172, 173) and preventing epigenetic changes through the use of mTOR inhibitors, inhibited the shift in metabolism toward glycolysis and the acquisition of H3K4me3 at key inflammatory gene promoters.

With regards to organ transplantation, Fadi Lakkis and colleagues described that monocytes are able to recall skin grafts exhibiting memory features normally attributed to adaptive immune cells. Using BALB/c Rag^−^/^−^ mice as recipients of BALB/c (H-2^d^), allogeneic B6 (H-2^b^) and “third-party” C3H (H-2^k^) donor skin grafts rechallenged with B6 splenocytes 1 week after engraftment, the study demonstrated that monocytes were able to mount an inflammatory response 1 week after transplantation independently of the adaptive immune system (134). Interestingly, BALB/c recipients mounted an allo-dependent response to allogeneic B6, but also to “third-party” C3H (134). Although the third-party response was statistically lower than the allo-dependent response, the data suggests that monocytes are able to respond to non-specific recall stimuli, a feature of trained immunity. Challenging the view of non-specific responses mediated by macrophages, studies from Xian Li and colleagues reported that reconstituted Rag^−^/^−^γc^−^/^−^ hosts with syngeneic B6 CD4+ T cells and donor BALB/c cells results in *in vivo* killing of donor BALB/c cells transferred 2 weeks after reconstitution but does not result in the rejection of “third-party” C3H cells (174). This argues in favor of further investigating epigenetic mechanisms of macrophage recall processes and the potential implication of SIRPα in these processes, as described above. Remarkably, this study demonstrated that macrophage-mediated rejection of recall responses can be prevented with CD40/CD40L costimulatory blockade during the first stimuli. This suggests that anti-CD40L mAb treatment may prevent the accumulation of memory-like macrophages in the donor allografts early after transplantation.

Inhibition of trained macrophages in the allograft can be achieved by targeting the mTOR pathway in myeloid cells *in vivo* (155). We recently demonstrated that vimentin promotes macrophage training *via* dectin-1 signaling, which results in increasing deposition of H3K4me3 at the promoter of TNF-alpha and IL-6 upon a secondary stimulation with HMGB1, another protein highly expressed in the donor allograft. The same trend in epigenetic changes occur *in vivo* using an experimental mouse model of heart transplantation. Interestingly, inhibition of trained immunity with mTORi-HDL nanobiologics promoted long-term allograft survival *via* Treg expansion and inhibition of cytotoxic T cells.

In addition to targeting trained immunity in organ transplantation *via* the administration of mTORi-HDL nanoparticles, there is the potential use of small molecules that inhibit epigenetic-related proteins including HDAC inhibitors (HDACi) and BET inhibitors (BETi). HDACi are thought to primarily inhibit histone deacetylation, thus promoting gene expression at specific loci, while BETi inhibit the binding of BET proteins to acetylated regions of the genome, which normally promote gene expression at specific loci (175). However, reports specifically implicating their use in the context of transplant have been few. In regards to the use of BET inhibitors, a synthetic compound, I-BET, was developed that was shown to repress gene expression of LPS-inducible genes in bone marrow derived macrophages (BMDM) *ex-vivo* (176). The importance of BET proteins in aiding gene expression of inflammatory genes in macrophages was established through use of *brd2 lo* mice and silencing of BET proteins through siRNA studies (177). With regards to the use of an HDACi to prevent allograft refection, an inhibitor of HDAC6, KA1010, was shown to reduce allograft skin rejection through mechanisms that involved reduction in CD4 T cells with an increase in the Treg population (178). The effect of HDACi on macrophages on the other-hand is not clear and *in-vitro* experiments on BMDM treated trichostatin A (TSA), a class I and II HDACi, displayed a phenotype favoring progenitor-like myeloid cells rather than differentiated macrophages. These macrophages displayed a mixed M1/M2 phenotype according to cytokine and chemokine secretion analysis, suggesting that treatment with HDACi alone may not be a suggestable mode of therapeutic treatment (179). On the contrary, a study by Thangavel and colleagues demonstrated that combinatorial treatment of TSA with 5-Aza 2-deoxycytidine (Aza), a DNA methyl transferase (DNMT) inhibitor, was able to promote an M2 phenotype in macrophages and to reduce inflammation in an acute lung injury model (180). Overall, while drugs targeting epigenetic modifiers including HDACs, BET proteins and DNMTs do hold promise as therapeutic approaches that promote long-tern allograft survival in organ transplantation, it appears that successful use of these drugs to prevent graft rejection will require their use to be in combination with other drugs.

## Concluding Remarks and Future Perspectives

Organ transplantation is a life-saving strategy for terminal and irreversible organ failure. While the solid organ transplantation has achieved an excellent success in short-term graft survival rates, the long-term survival rates of organ transplants remain suboptimal. The pathophysiology of graft rejection is multifactorial and growing evidence suggests that macrophages are key mediators of acute and chronic graft loss, through the secretion of inflammatory mediators that activate the adaptive alloimmune response. Historically, accumulation of macrophages in the donor organ has been associated with transplant rejection (181, 182) as allogeneic antigen-primed macrophages mediate allograft rejection (183). However, not all macrophages are associated with graft loss. Different subpopulations of macrophages regulate the allograft immune response through protective mechanisms based on their phenotype and function. As a result, the identification of the *in vivo* signaling pathways that govern macrophage polarization and modulate their function may provide new therapeutic targets that promote allograft survival.

Therapeutic agents that regulate macrophage polarization that promote the accumulation of regulatory macrophages are potential candidates to promote long-term allograft survival in transplant recipients. In addition, identification of previously unrecognized pathways associated with chronic allograft rejection may offer new therapeutic avenues for intervention. Classically, the innate immune response has been defined as a non-specific rapid response, followed by a later-onset of antigen-specific adaptive immune cells. However, accumulating findings have challenged the fact that innate immune cells do not possess a memory, leading to the concept of innate immune memory and trained immunity. This concept postulates that stimulated innate immune cells are primed to recognize specific ligands and secrete specific cytokines more rapidly upon a second stimulus. This type of memory is retained by mechanisms of epigenetic and metabolic changes in innate immune cells exposed to particular ligands. As a result, therapeutic targeting of trained immunity represents a novel treatment paradigm to prevent allograft rejection. Thus, a comprehensive understanding of the immunobiology of different macrophage subsets is crucial to develop novel strategies that promote long-term allograft survival in transplant recipients and to translate macrophage-targeted therapeutic strategies in the clinic.

## Author Contributions

All authors contributed to the article and approved the submitted version.

## Funding

The authors**’** work is supported by National Institutes of Health grants R01 AI139623AI (JO), and NIH-T32CA078207 (FO).

## Conflict of Interest

The authors declare that the research was conducted in the absence of any commercial or financial relationships that could be construed as a potential conflict of interest.
